# Altered Lipid Levels in Untreated Patients with Early Polymyositis

**DOI:** 10.1371/journal.pone.0089827

**Published:** 2014-02-24

**Authors:** Han Wang, Yingying Cai, Lin Cai, Yingchun Hu, Xianxiang Chen, Juelin Deng

**Affiliations:** 1 Cardiovascular Disease Research Institute, The Third People’s Hospital of Chengdu, The Second Affiliated Chengdu Clinical College of Chongqing Medical University, Chengdu, Sichuan, People’s Republic of China; 2 Department of Surgery, Renmin Hospital of Hubei University of Medicine, Shiyan, Hubei, People’s Republic of China; 3 Department of Geriatrics, West China Hospital, Sichuan University, Chengdu, Sichuan, People’s Republic of China; National Institutes of Health, United States of America

## Abstract

**Background:**

Little is known so far on the lipid profile in polymyositis (PM) patients. Our aim is to identify lipid profiles in untreated patients with early PM, to assess the association between lipid profiles and C-reactive protein (a sensitive marker of inflammation) in these patients.

**Methods and Findings:**

This work was conducted as a case-control study. Sixty untreated patients with PM and 60 age- and sex-matched healthy controls were included. The duration of PM was less than six months, and none of them had received intermittent or regular corticosteroids or disease-modifying antirheumatic drugs or biological agents prior to the study. Triglyceride (TG), total cholesterol (TC), LDL-cholesterol (LDL-C), and HDL-cholesterol (HDL-C), and C-reactive protein (CRP) were assessed using standard techniques. Thirty patients (50%) had a decreased level of HDL-C and 47% had an increased level of TG. The levels of HDL-C, LDL-C, and TC in PM were significantly lower than in controls (*P*<0.001, *P*<0.01, *P*<0.001, respectively). The level of TG was significantly higher in PM than in controls (*P*<0.001). The level of very low LDL-cholesterol (VLDL-C), and the ratios of VLDL-C/LDL-C, TC/HDL-C, and LDL-C/HDL-C were significantly higher than in controls (all *P*<0.001). Serum CRP levels correlated negatively with HDL-C (*r* = −0.352, *P* = 0.006) and TC (r = −0.262, P = 0.043). After adjustment for age, gender, smoking, drinking, body mass index, and pulmonary fibrosis/infection, linear regression model demonstrated that CRP is associated with HDL-C among PM patients (*P* = 0.028).

**Conclusions:**

Dyslipidemia is a common feature in patients with PM that is characterized by a decrease in HDL-C and an increase in TG, suggesting a high risk of atherosclerosis. The Inflammatory condition in PM may account for the metabolism of HDL-C.

## Introduction

Altered lipid levels have been reported in patients with autoimmune disorders [1]–[3]. For example, patients with active untreated rheumatoid arthritis (RA) have reduced HDL-cholesterol (HDL-C), total cholesterol (TC), and LDL-cholesterol (LDL-C) [1]. Decreased HDL-C and raised triglyceride (TG) levels, namely the “lupus pattern of dyslipoproteinemia”, have also been found in active patients with systemic lupus erythematosus (SLE), where inflammation and disease activity may be related to the changes of lipid levels [3]. These observations showed an interaction between lipoproteins alteration (quantitatively and/or qualitatively) and inflammatory metabolism, and furthermore, suggested a close link between autoimmune disorders and atherosclerosis, due to traditional and nontraditional risk factors for cardiovascular diseases [1]–[3].

Previous studies have reported abnormal lipid profiles in patients with dermatomyositis [4]–[6]. These results demonstrated that dyslipidemia is a common feature in dermatomyositis patients which is characterized by an increase in TG and a decrease in HDL-C, suggesting a high risk of atherosclerosis in dermatomyositis. Polymyositis (PM), together with dermatomyositis, belongs to idiopathic inflammatory myositis, which represents a heterogeneous group of autoimmune systemic diseases characterized by chronic muscle weakness and inflammatory cell infiltrates in skeletal muscle [7]. Similarly, cardiovascular disease is also one of the leading causes of death in PM [8]. In fact, the cardiovascular risk in PM is increased in comparison the general population. For example, Tisseverasinghe et al. found an increased incidence of arterial events associated with hypertension (adjusted RR 2.6, 95% CI 1.2–5.5) and lipid disorders (adjusted RR 2.6, 95% CI 1.0–6.5) in dermatomyositis and PM from Canada administrative databases [9]. Altered lipid level, such as increasing serum cholesterol/TG levels, and low HDL-C, is closely associated with cardiovascular disease in the general population and patients with autoimmune disorders including PM [10]. However, the association between lipid levels and cardiovascular disease risk in autoimmune disorders may be more complex than that in the general population, because systemic inflammation, such as C-reactive protein, may contribute to changes of lipid levels [10].

Until now, to our best knowledge, detailed investigations of serum lipid profiles in patients with PM have not yet to be performed. What is more, there is currently scarce information regarding the role of acute phase reactants on lipid metabolism of PM. Therefore, we conducted a case-control study to compare lipid levels in untreated early PM patients with those in healthy individuals, and to assess the relationship of the inflammatory condition in early PM with lipid profiles.

## Materials and Methods

The study was approved by the ethic committee of the Third People’s Hospital of Chengdu. Written informed consent was obtained from subjects enrolled.

This was a case-control study. We studied sixty PM patients (mean age ± SD 42.9±12.5, range 18–70 years, 44F) referred to the cardiology department of the No 3 Hospital of Chengdu and the rheumatology department of West China Hospital between September 2009 and February 2013. All patients were affected PM according to the criteria of Bohan and Peter by skilled clinicians [11], with a duration less than six months (mean time ± SD 3.3±1.8, range 1–6 months). Sixty healthy individuals with similar age and sex (mean age ± SD 42.9±11.7, range 19–68 years, 44 F) were recruited as a control group. Patients with diseases that may cause secondary hyperlipidaemia including chronic kidney disease, diabetes mellitus, nephritic syndrome, hypothyroidism and obstructive liver disease, as well as other connective tissue diseases, such as SLE, RA and mixed connective-tissue disease, were excluded. In addition, none of the PM patients had received intermittent or regular corticosteroids, disease-modifying antirheumatic drugs or biologics before serum sampling; and none of the PM patients had a history of heart diseases or interventional management for cardiovascular diseases.

Patients who agreed to participate were scheduled for an additional appointment. Information, such as general data, physical examination, and blood sample, on each patient was collected through face-to-face interviews conducted by trained medical students or clinical doctors with a self-report questionnaire. Clinical information such as respiratory tiredness, myalgia, muscle weakness, arthralgia, and dysphagia were collected according to the description of patients, and myoedema was confirmed by muscle biopsy. Body mass index (kg/m^2^) was calculated as body weight (kilograms) divided by height (meters squared). Patients were asked whether they had ever had habits of smoking, and alcohol consumption. Smokers or drinkers were defined as a subject who had been regularly smoking or drinking for at least one year and was still smoking or drinking, whereas nonsmokers or nondrinkers were defined as those who had not [12]. In addition, High-resolution CT was used to assess lung involvement. In the study, lung involvement included pulmonary infection, pulmonary fibrosis, pulmonary tuberculosis, and pleural effusion.

After an overnight fast, venous blood was taken in the morning from all cases. The following laboratory parameters were assessed: TG (normal range: 0.29–1.83 mmol/L), total cholesterol (TC) (normal range: 2.8–5.7 mmol/L), LDL-cholesterol (LDL-C) (normal range: <4.0 mmol/L), HDL-C (normal range: >0.9 mmol/L), and C-reactive protein (CRP) (normal range: <5.0 mg/L). Non-HDL-cholesterol (Non-HDL-C) and VLDL-cholesterol (VLDL-C) values were calculated according to the following formulas: Non-HDL-C = TC - HDL-C; VLDL-C = TG/5. Atherogenic index (TC/HDL-C ratio), VLDL-C/LDL-C, and LDL-C/HDL-C ratio were calculated.

Statistical analysis was performed using SPSS 16.0 for Windows (SPSS, Chicago, USA). Mean and standard deviation (S.D.) for quantitative variables and number of patients (%) for categorical variables were calculated. All quantitative variables are normally distributed, and comparisons of them between two groups were performed by two-tailed paired Student’s *t*-test. Corrections between variables were analyzed using Pearson’s correlation test. The association between CRP and HDL-C level was controlled for age, gender, smoking, drinking, body mass index, and pulmonary fibrosis/infection in a linear regression model. A *p*-value of less than 0.05 (two-tailed) was considered statistically significant.

## Results

The baseline characteristics of patients are outlined in Table 1. The frequency of elevated TG, elevated TC, elevated LDL-C, reduced HDL-C, and CRP positivity were 47%, 5%, 0%, 50%, and 60%, respectively.

**Table 1:** Clinical characteristic of untreated patients with early polymyositis and controls.

Clinical characteristic	Patients (n = 60)	Controls (n = 60)
Age (years)	42.9±12.5	42.9±11.7
F/M	44/16	44/16
Smoking (n, %)	6/60 (10)	7/60 (10)
Drinking (n, %)	5/60 (8.3)	6/60 (10)
Body mass index (kg/m^2^)	22.7±2.8	22.1±2.6
systolic pressure (mmHg)	117.1±11.3	118.2±10.5
diastolic pressure (mmHg)	75.7±6.2	74.9±7.5
Respiratory tiredness (n, %)	7/60 (12)	0/60 (0)
Myalgia (n, %)	45/60 (75)	0/60 (0)
Muscle weakness (n, %)	45/60 (75)	0/60 (0)
Myoedema (n, %)	13/60 (22)	0/60 (0)
Arthralgia (n, %)	22/60 (37)	0/60 (0)
Raynaud's phenomenon (n, %)	9/60 (15)	0/60 (0)
Dysphagia (n, %)	6/60 (10)	0/60 (0)
Pulmonary fibrosis/infection (n,%)	29/60 (48)	NA
Positive ANA (n, %)	44/60 (73)	NA
Positive anti-Jo-1 antibody (n, %)	7/60 (12)	NA
Elevated triglyceride (n, %) normal range: 0.29–1.83 mmol/L	28/60 (47)	12/60 (20)*
Elevated total cholesterol (n, %) normal range: 2.8–5.7 mmol/L	3/60 (5)	5/60 (8)
Elevated LDL-cholesterol (n, %) normal range: <4.0 mmol/L	0/60 (0)	4/60 (7)
Reduced HDL-cholesterol (n, %) normal range: >0.9 mmol/L	30/60 (50)	0/60 (0)*
Positive CRP (n, %) normal range: <5.0 mg/L	36/60 (60)	4/60 (7)*
CRP levels	11.6±13.9	3.3±1.2*

F, female; M, male; CRP, C-reactive protein; HDL-C, high-density lipoprotein -cholesterol; LDL-C, low-density lipoprotein- cholesterol; NA, not available.

* P<0.05.

Table 2 shows the lipid profiles of PM patients and healthy individuals. In PM patients, levels of TG, VLDL-C, total/HDL cholesterol ratio, VLDL/LDL cholesterol ratio and LDL/HDL cholesterol ratio were higher than those of healthy individuals (*P*<0.001, 1.89±0.81 mmol/L vs 1.30±0.67 mmol/L; *P*<0.001, 0.38±0.16 mmol/L vs 0.26±0.13 mmol/L; *P*<0.001, 4.54±1.20 vs 3.18±0.89; *P*<0.001, 0.18±0.10 vs 0.10±0.05; *P*<0.001, 2.62±0.79 vs 1.83±0.68, respectively), while HDL-C, TC and LDL-C levels in PM patients were lower than those of controls (*P*<0.001, 0.93±0.27 mmol/L vs 1.54±0.31 mmol/L; *P*<0.001, 4.05±0.94 mmol/L vs 4.70±0.74 mmol/L, and *P*<0.05, 2.35±0.69 mmol/L vs 2.69±0.72 mmol/L, respectively). There were not significantly different for non-HDL-cholesterol between the patients and the healthy individuals.

**Table 2:** Lipid profiles in untreated patients with early polymyositis and controls.

	Patients(n = 60)	Controls(n = 60)	P
Triglyceride (mmol / L)	1.89±0.81	1.30±0.67	<0.001
Total cholesterol (mmol / L)	4.05±0.94	4.70±0.74	<0.001
LDL-cholesterol (mmol / L)	2.35±0.69	2.69±0.72	<0.01
HDL-cholesterol (mmol / L)	0.93±0.27	1.54±0.31	<0.001
Non-HDL-cholesterol(mmol / L)	3.11±0.83	3.16±0.82	NS
VLDL-cholesterol (mmol / L)	0.38±0.16	0.26±0.13	<0.001
VLDL/LDL cholesterol ratio	0.18±0.10	0.10±0.05	<0.001
Total/HDL cholesterol ratio	4.54±1.20	3.18±0.89	<0.001
LDL/HDL cholesterol ratio	2.62±0.79	1.83±0.68	<0.001

HDL-C, high-density lipoprotein -cholesterol; LDL-C, low-density lipoprotein- cholesterol; NS, not significant; VLDL-C, very low LDL-cholesterol.

The correlations between lipid profiles and CRP in PM patients have been seen in Table 3. Serum CRP levels correlated negatively with HDL-C (*r* = −0.352, *P* = 0.006) and TC (r = −0.262, P = 0.043).

**Table 3:** Correlation between CRP and lipid profiles among untreated patients with early polymyositis.

	CRP r p
TC	−0.262 0.043
TG	−0.070 0.593
LDL-c	−0.179 0.171
HDL-c	−0.352 0.006
Non-HDL	−0.181 0.167
VLDL	−0.069 0.601
TC/HDL-c	0.165 0.207
LDL-c/HDL-c	0.199 0.128
VLDL-c/LDL-c	0.027 0.836

HDL-C, high-density lipoprotein -cholesterol; LDL-C, low-density lipoprotein- cholesterol; NS, not significant; VLDL-C, very low LDL-cholesterol.

We assessed the association between HDL-C and CRP in a linear regression model (Table 4). After adjustment for age, gender, smoking, drinking, body mass index, and pulmonary fibrosis/infection, multiple linear regression models demonstrated that CRP is associated with HDL-C among PM patients (P = 0.028).

**Table 4:** The association between C-reactive protein and high-density lipoprotein - cholesterol among untreated patients with early polymyositis in a linear regression model.

	B	SE	Beta	t	p
Constant	1.749	0.309		5.659	0.000
Age	−0.002	0.003	−0.074	−0.573	0.569
Gender	0.070	0.084	0.115	0.833	0.409
Smoking	−0.089	0.113	−0.099	−0.791	0.432
Drinking	−0.155	0.122	−0.159	−1.274	0.208
Body mass index	−0.029	0.015	−0.291	−1.970	0.054
Pulmonary fibrosis/infection	−0.048	0.087	−0.089	−0.720	0.475
C-reactive protein	−0.006	0.002	−0.287	−2.265	0.028

## Discussion

In the present study, untreated patients with early PM showed a frequent dyslipidaemia and had a more marked atherogenic lipid profiles compared with the healthy individuals. These atherogenic lipid profiles include decreased HDL-C, increased TG, VLDL-C, total/HDL-C ratio, VLDL/LDL-C ratio, and LDL/HDL-C ratio, which are thought to be risk factors for cardiovascular diseases, contributing to accelerated atherosclerosis not only in the general population, but also in SLE and RA [1], [3]. In addition, serum CRP, an independent risk factor for cardiovascular diseases [10], has been found to be associated with HDL-C in the population.

To our knowledge, it is the first study on serum lipid profiles in untreated early PM patients. Recent studies focusing on lipid profiles in dermatomyositis have addressed a pattern of dyslipidemia dyslipoproteinemia characterized by an increase in TG and a decrease in HDL-C [4]–[6], which are also similar to those seen in patients with metabolic syndrome and other autoimmune diseases. Analogous to these studies, a consistent pattern of low HDL-C and raised TG levels has been seen in our patients compared with age- and sex-matched controls, but it becomes more complex with respect to TC and LDL-C levels. Some trials focusing on RA showed significant elevations in these parameters relative to controls but other do not [1]. In the present study, levels of TC and LDL-C are significantly lower in patients with early PM than controls. It is very interesting that the results are comparable to the findings in severe, untreated, active RA [7]. For example, TC and LDL-C levels often decline in patients with early active RA, as well as in other pathologies/conditions associated with inflammation or infection, such as severe meningococcal sepsis, trauma or cancer [13]–[15], and these changes are also frequently in line with inflammatory marker elevations including CRP. Similarly, there is a negative correlation between TC and CRP in our study. Therefore, TC and LDL-C levels appear normal or even low in these patients possibly due to different inflammatory condition and/or stages of disease. Although the exact mechanisms remain unclear, cytokine-induced activation of the reticuloendothelial system may be potentially critical to such changes [7], as recent studies in activated macrophages have shown that the activation of reticuloendothelial system usually occurs with inflammation and lowers LDL-C [16]. It is noted that low TC level is often associated with increased death, which may suggest a poor prognosis in these patients [17]. In addition, we found an unfavorable atherogenic index (TC/HDL cholesterol ratio) similar to findings in RA. This ratio is more stable than the different individual lipid profiles including TC, LDL-C and HDL-C, and it does not change dramatically with treatments in many autoimmune diseases [7].

In the present study, we found a close association between CRP and HDL-C after controlling for age, gender, smoking, drinking, body mass index and pulmonary fibrosis/infection. In fact, low HDL-C in autoimmune diseases including RA often negatively correlated with acute phase reactants including CRP and erythrocyte sedimentation rate, and further results also showed that these acute phase reactants are at least partially responsible for the presence of low HDL-C in RA [18]. The precise mechanisms remain to be established, however, inflammatory cytokines and acute phase reactants may act in adipose tissue, skeletal muscle and the liver to promote metabolic changes that result in lipoproteins abnormalities [19]. A number of enhanced productions of other acute phase reactants, such as tumor necrosis factor-alpha (TNF-alpha), interleukin-1 (IL-1) and IL-6, may also play an important role in lipoproteins alteration in untreated patients in early PM, as well as in other diseases [20], [21]. On the other hand, anti-inflammatory therapy may lead to increases, not only levels of HDL-C, but also TC, LDL-C and, perhaps, TG. There are also increasing data showing that lipid-lowering therapy positively impacts inflammation [22], [23], suggesting an interaction between lipid profiles and inflammation in autoimmune diseases. Inflammation may be the driving force of dyslipidemia, and it might be that normalizes during corticosteroids administration. However, it’s should be noted on one hand corticosteroids can reduce inflammation; on the other hand they have cardiovascular adverse events.

Other mechanisms may be proposed to explain the altered lipid levels in PM. For example, lipoprotein lipase (LPL), a key enzyme in lipid metabolism, can influence the lipid profiles and induce lipid peroxidation, resulting in enhanced clearance of HDL-C and reduced levels of HDL-C [24]. Therefore, patients with impaired activity of LPL might have relatively low HDL levels, as well as high TG, high VLDL, and low LDL levels, similar to the results observed in our populations. In fact, a higher VLDL-C/LDL-C ratio often suggests the impaired activity of LPL [25]. In the present study, there is a higher VLDL-C/LDL-C ratio compared with healthy individuals (0.18±0.10 vs. 0.10±0.05), suggesting LPL activity may be impaired in patients with PM. Other mechanisms, such as immobilization, and disease-specific antibodies, may also be involved.

The current study has some limitations that deserve a mention. Firstly, the sample of PM patients was relatively small. However, as differences between PM patients and controls for the outcome measures were generally large with limited variability, even small differences were detectable within the bounds of statistical significance. Secondly, PM might be an overdiagnosed entity [26]–[28]. However, the criteria of Bohan/Peter, as a clinical standard, is widely used in the world. Thirdly, pulmonary infections in several PM patients could be contributing to elevated CRP levels as a confounder, however, the result remained unchanged when we controlled for the pulmonary infections in linear regression model.

In the current study, untreated patients with early PM may have reduced HDL-C and raised TG levels, and often have an unfavorable atherogenic index, suggesting a high risk of atherosclerosis and cardiovascular diseases. In the early stage of PM, TC and LDL-C levels are also low in untreated PM patients and TC levels inversely correlated with CRP. Therefore, physicians should be aware of that the higher risk of cardiovascular diseases may have already presented in patients at the time of initial diagnosis of PM. However, more research is needed including association between these lipid profiles and cardiovascular endpoints in PM.
